# Huangkui capsule mitigates diabetic nephropathy via epigenetic therapy effects

**DOI:** 10.3389/fphar.2026.1775173

**Published:** 2026-02-24

**Authors:** Yihong Yu, Haitao Tang, Nan Li, Haitao Ge, Jie Wu, Harvest F. Gu

**Affiliations:** 1 Laboratory of Molecular Medicine, China Pharmaceutical University, Nanjing, China; 2 Laboratory of Minigene Pharmacy, China Pharmaceutical University, Nanjing, China; 3 College of Pharmacy, Chemistry and Chemical Engineering, Taizhou University, Taizhou, China; 4 Department of Endocrinology, Jiangsu Province Hospital of Chinese Medicine, Nanjing, China; 5 School of Chinese Medicine, Nanjing University of Chinese Medicine, Nanjing, China; 6 School of Pharmacy, Qilu Medical University, Zibo, China

**Keywords:** Abelmoschus manihot (L.), diabetic nephropathy, DNA methylation, epigenetic pharmacology, transcriptome sequencing, whole genome bisulfite sequencing

## Abstract

**Background:**

Huangkui capsule (HKC), a Chinese herbal medicine derived from *Abelmoschus manihot* (L.) ethanol extract, has clinical efficacy against diabetic nephropathy (DN). Our research group has actively engaged in exploring the efficacy of HKC in treating DN. The underlying pharmacological mechanisms have progressively become clearer but its epigenetic mechanisms remain unclear.

**Objective:**

To elucidate HKC’s epigenetic role in the treatment of DN.

**Methods:**

Db/db mice (a type 2 diabetes/DN model) were orally administered HKC or vehicle for 4 weeks. Kidney tissues underwent whole-genome bisulfite sequencing and transcriptome profiling to assess DNA methylation and gene expression patterns.

**Results:**

HKC significantly reduced urinary albumin/creatinine ratios, indicating renal protection. Comparative methylation analysis revealed HKC regulated the distribution of 5 mC by modulating *Tet2* expression, thereby influencing abnormal methylation patterns in DN. Integrative analysis identified 12 DN-associated genes with reversed methylation and expression post-HKC treatment, including *Cdk8, Pde4d, Pisd-ps3*, and *Zc3h7a*, which showed high susceptibility to DN progression and HKC intervention. Functional annotation linked these genes to immune regulation, synaptic signaling, and Notch pathways.

**Conclusion:**

This study provides the first evidence that HKC ameliorates DN through epigenetic therapy effects, specifically by restoring DNA methylation and transcriptional activity of renal target genes. Further biological experiments to validate these findings are necessary.

## 1 Introduction

Diabetes, a chronic metabolic disorder, affects approximately 537 million adults worldwide, accounting for 10.5% of individuals aged 20 to 79. Among them, with over 90% were diagnosed with type 2 diabetes (T2D) (Sun et al., 2022). Diabetic nephropathy (DN) is the most prevalent microvascular complication, occurring in approximately 40% of T2D patients (Mark et al., 2023). Epidemiological data indicate that the incidence of DN is rising alongside increasing diabetes prevalence (Chu et al., 2024). As a leading cause of end-stage renal disease (ESRD), DN accounts for 35%–50% of ESRD cases, imposing a significant economic burden on both individuals and society.

In recent years, genetic and epigenetic studies have been conducted to explore the pathogenesis of DN (Gu, 2019b; Gu, 2019a; Sandholm et al., 2023). Genetics focuses on genetic variation in nuclear and mitochondrial DNA sequence, while epigenetics examines mechanisms by which genetic information related to traits is preserved and passed on through processes like DNA methylation, histone modification, and non-coding RNA regulation, without changes to the DNA sequence itself. Accumulating evidence has demonstrated that DNA methylation has significant involvement in numerous biological processes, such as maintaining immune homeostasis, renal tubular antioxidant defense, protection of podocyte mitochondrial function, and anti-apoptotic mechanisms associated with transcriptional regulation (Gu, 2019b; Gu, 2019a; Sandholm et al., 2023; Chen et al., 2024). Thus, DNA methylation analysis not only enhances our understanding of the epigenetic mechanisms involved in DN pathogenesis but also provides insights into the pharmacological actions of drugs used to treat DN.

Huangkui capsule (HKC), as a traditional Chinese patent medicine, has been used to treat renal diseases, including DN (Li et al., 2021). HKC is made from the ethanol extract of *Abelmoschus manihot* (L.) and received approval from the China Food and Drug Administration (Z19990040) in 1999 (Guo et al., 2015; Li et al., 2021). Similar to the discovery story of Artemisinin (Shi et al., 2022), the medical application of *A. manihot* (L.) was first recorded in the Handbook of Prescriptions for Emergencies by Mr. Hong Ge in the Eastern Jin Dynasty (317–420 AD), China. Previously, clinical studies have demonstrated the effective improvement of HKC in nephritis, chronic kidney disease, and IgA nephropathy (Zhang et al., 2014; Li et al., 2017; Li et al., 2020). In 2022, Zhao et al. conducted a multicenter, randomized, double-blind, parallel-controlled clinical trial and reported that HKC administration is an effective therapy for reducing albuminuria and proteinuria in T2D patients with DN (Zhao et al., 2022b). Over the last 5 years, our research group has put efforts into exploring the pharmaceutical mechanism of HKC in the treatment of DN, using db/db mice as a model for study of T2D and DN (Sharma et al., 2003). We have investigated the therapy effects of HKC on the gut-kidney axis by using NOD and db/db mice, the animal models for study of type 1 and type 2 diabetes respectively. (Wu C. et al., 2022). In parallel, we systematically identified the main constituents of HKC and their metabolites in the serum, intestinal contents, urine, kidney, heart, liver, jejunum, and colon tissues of db/db mice following oral administration by using HPLC-Q-TOF-MS/MS analytical approach (Diao et al., 2023). We also demonstrated that HKC has pharmacological efficacy in the regression of the development of DN via the regulation of solute carriers in proximal and distal convoluted tubules of kidneys (Yu et al., 2023a; Yu et al., 2023b). Furthermore, we have carried out single-cell and spatial RNA sequencing analyses of kidneys in db/db mice to predict the cell-specific targets, to elucidate the heterogeneity of mitochondrial damages, and found the key receptors and regulators responded by HKC (Wu et al., 2023a; Wu et al., 2023b; Wu et al., 2024). All these studies, however, have no consensus on the epigenetic effects of HKC in the treatment of DN.

In the present study, we conducted whole-genome bisulfite and transcriptome sequencing analyses to better understand the kidney target genes of HKC in the treatment of DN. First, we assessed the genome-wide DNA methylation levels in DN, followed by analyzing the DNA methylation changes in db/db mice after HKC administration. We then performed the renal bisulfite and transcriptome sequencing analyses. Thereby, the present study could provide novel insights to explore the epigenetic pharmacological effects of HKC in treating DN.

## 2 Materials and methods

### 2.1 Animal management and drug administration

The db/db (BKS.Cg-Dock7m^Lepr +/+ db/J^) and nondiabetic control (C57BL/6J) male mice at the age of 8 weeks were purchased from the Institute of Model Animal Research of Nanjing University, Nanjing, China. The mice were housed in the specific pathogen-free barrier environment of the Animal Experimentation Center, Xuanwu Campus, China Pharmaceutical University (CPU), and given adequate sterile drinking water and standard chow. The temperature was 25 °C ± 2 °C and the relative humidity was 40%–70% in the animal room. All mice were acclimatized for 1 week before the experiments were conducted. All experiments with the animals were approved by the Animal Ethics Committee of CPU and conducted according to the relevant experimental regulations.

As we previously reported (Diao et al., 2023; Yu et al., 2023b), DN in db/db mice was identified based on the presence of body glucose (BG) > 16.7 mmol/L and the urine micro-albuminuria to creatinine ratio (UACR) > 200 mg/g on two consecutive tests. The db/db mice with high urinary proteinuria were then randomly divided into HKC and DN groups. In HKC group, the suspension of HKC (0.84 g/kg/day, dissolved in distilled water) was freshly prepared and intragastrical administered to the db/db mice, while the equivalent distilled water was used simultaneously in DN group. The treatment of HKC in DN was carried out once a day for 4 weeks. HKC was purchased from Suzhong Pharmaceutical Group, Co., Ltd., Taizhou, China. The db/db mice with high BG but low UACR (db/db mice with no or minimal proteinuria) were grouped as T2D. BG (mmol/L), body weight (BW) (g), dietary intake (g), water intake (mL), and urine output (mL) were examined every week. By using a metabolic cage (Fengshi Laboratory Animal Equipment Co., Ltd., Suzhou, China), urine samples were collected for 12 h. Urine from db/db mice was collected weekly to examine UACR and urine albumin excretion rate (UAER). Quantitative ELISA kits (Elabscience, Wuhan, China) were used to measure albuminuria (MAU) and creatinine (Cr) levels in urine samples, and to calculate UACR (MAU/Cr) and UAER (MAU/12 h) values. After 4 weeks of HKC treatment, experimental mice were euthanized by decapitation following intraperitoneal administration of 30 mg/kg sodium pentobarbital. Kidney tissue samples were harvested and frozen in liquid nitrogen for subsequent experiments.

### 2.2 Histopathological examination of kidneys

The kidneys were removed by cardiac perfusion with phosphate buffered saline and placed in formaldehyde tissue fixative. The fixed kidney tissues were embedded in paraffin and the blocks were sectioned at 4 μm with HistoCore Bio-Cutter (Leica Biosystem, Germany). The sections were then stained with Hematoxylin and Eosin (H&E) (BASO, Zhuhai, China) and/or Periodic Acid-Schiff (PAS) staining solution (Aifang Biological, Hunan, China) according to the standard procedures and finally mounted on a CX23 light microscope (Olympus, Japan) for analysis. As we have previously reported (Wu C. et al., 2022), in the present study, H&E and PAS staining sections from each animal were analyzed for semi-quantification of glomerular area, ratio of vacuolar and staining area by using the software of Image-Pro Plus (version 6.0.0260).

### 2.3 DNA library preparation and whole genome bisulfite sequencing

Genomic DNA was extracted from the kidney tissues by using the animal genomic DNA kit (E.Z.N.A.® Tissue DNA Kit, Omega Bio-Tek, United States). DNA integrity of extractions was inspected by using 1% agarose gel electrophoresis. The bisulfite transformation and DNA fragment purification were done by using a transformation kit (EZ DNA Methylation-Gold Kit, Zymo Research, United States). A high-throughput sequencing analysis was subsequently carried out with Illumina HiSeq 2500. After removing unknown nucleotides and low-quality read lengths from the original read lengths, the trimmed clean reads were detected.

For whole genome bisulfite sequencing (WGBS) analysis, the raw Fastq files obtained were trimmed by Trimmomatic (v0.39–2) under the conditions of allowing up to two base mismatches, a sliding window size of 4, and an average quality threshold of 15, and clean reads were generated by discarding unpaired reads and paired reads with a final length of less than 75 bp. The clean reads were compared to the mouse genome (mm10) by Bismark (v0.24.0) to generate bam files and extract methylation information. After removing loci with zero coverage, the following steps were performed using MethylKit (v1.22.0): methylation levels in the whole genome were calculated using the sliding window method (2 kb), as well as the Pearson correlation coefficients of all the samples (Akalin et al., 2012) to check the reproducibility within the administered groups and compare methylation differences between groups; the “methylation level” was determined by the fraction of methylated cytosines, i.e., the proportion of methylated cytosines to all cytosine sites in the region; the resulting clean reads were mapped to the mouse reference genome by using the Bismark software (v2.90); and the R package Methylkit (Wu et al., 2021) was used to estimate the methylation of CpG sites, promoter regions, CpG island region and gene annotation methylation status and ratios, as well as to identify differentially methylated regions (DMR) and differentially methylated site (DMS) between groups; 150 bp sliding-window regions with p-value <0.05 (with a step of 50 bp to satisfy an average coverage of reads greater than 10) were taken as the final DMR; base sites with q-values <0.01 and with a ratio of differences in methylation levels between base site groups of more than 25% of the base loci were DMS. DMR and DMS that overlapped with gene bodies or 2 kb regions upstream or downstream of the body region were considered as differentially methylated genes (DMGs), and genes with meth. diff >0 were categorized as hypermethylated genes (hyper genes), and genes with meth. diff <0 as hypomethylated genes (hypo genes).

### 2.4 RNA library preparation and transcriptome sequencing

RNAs were extracted from crushed kidney tissues with Trizol (Invitrogen, Carlsbad, United States), and total RNA integrity was detected by 1.2% agarose gel electrophoresis (meeting 28S rRNA/18S rRNA = 2.0). RNA concentration and purity were determined by Nanodrop 2000 UV spectrophotometer (A260/A230 = A260/A280 and >1.8), and RNA integrity was detected by Agilent 2100 (RIN value >9.0). Total RNA that met the criteria was reverse transcribed to cDNA, and ultrasonically sheared for Illumina library preparation (Illumina Truseq RNA sample prep Kit, Illumina, United States). After the library quality testing, the qualified libraries were put onto the Illumina Hiseq platform for PE150 sequencing.

For RNA transcriptome sequencing (RNA-Seq), the raw reads were trimmed as same as the procedure in the WGBS section. Trimmed data were aligned to the mouse genome (mm10) by Hisat2 (v2.2.1) to generate sam and bam files, the bam files were transformed to the gene expression count expression matrix by featurecount (v2.0.1). The expression matrices were analyzed for differential expression using DESeq2 software (v3.17), and genes with |log2FC| ≥ 0.5 and p-value <0.05 were identified as differentially expressed genes (DEGs). Among these, the genes with log2FC > 0 were categorized as upregulated genes (Up genes), while genes with log2FC < 0 were classified as downregulated genes (Down genes). Finally, the bam data were processed through rmats (v4.1.2) to output alternative 3′splice sites (A3SS), alternative 5′-splice sites (A5SS), mutually exclusive exons (MXE), retained intron (RI), and exon skipping (SE) types of variable shear events and generate sashimi-plot.

### 2.5 Verification of DNA methylation-associated gene expression

Conversion of RNA (removal of DNA) to cDNA using HiScript® III 1st Strand cDNA Synthesis Kit (Vazyme, Nanjing, China), three to four samples from each group were chosen for the experiment and RT-qPCR was carried out using Bio-Rad CFX connect™ Real-Time PCR Detection system (Bio-RAD, Singapore) and ChamQ SYBR Color qPCR Master Mix (Vazyme, Nanjing, China). The reaction system configuration and reaction conditions were performed according to the instructions. The primers were designed by the NCBI Primer-BLAST website and synthesized by Shanghai Biotech Co., Ltd., Shanghai, China (Supplementary Table S1).

### 2.6 Prediction of the GO and KEGG pathways

To explore the function of the genes, Gene ontology (GO) and Tokyo Encyclopedia of Genes and Genomes (KEGG) pathway enrichment analyses were performed using clusterProfiler (Wu et al., 2021). The pathways with P-value <0.05 were considered significantly enriched.

### 2.7 Statistical analysis

All quantitative results were expressed as mean ± standard error of the mean (SEM). All statistical analyses and graphics were performed using SPSS 22.0 software (SPSS, Chicago, IL) and R software (v4.3.1). Differences between groups were compared using unpaired Student’s t-test or one-way ANOVA test. The level of significance was presented as ∗ *P* < 0.05 and ∗∗ *P* < 0.01. Correlation analysis was performed by the Pearson correlation coefficient method. *P* < 0.05 were considered statistically significant.

## 3 Result

### 3.1 Physiological indexes and histopathological examination

**FIGURE 1:**
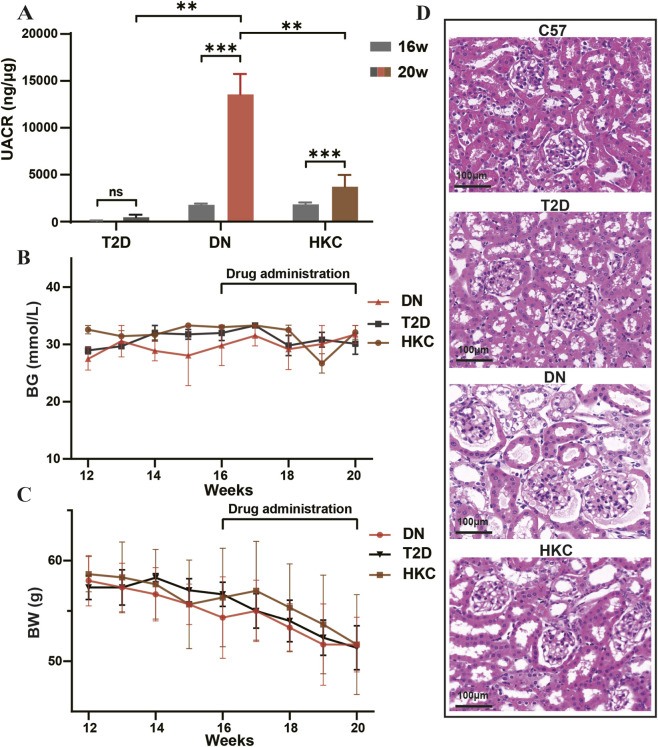
UACR reduction after HKC administration. **(A)** UACR values in DN group were higher than what in T2D group before administration (16w) but reduced after administration of HKC for 4 weeks (20w). **(B,C)** The levels of body glucose (BG) and body weight (BW) from 12w to 20w. **(D)** H&E staining kidney tissue sections demonstrating glomerular changes (scale bar, 100 μm). T2D: type 2 diabetes; DN: diabetic nephropathy; HKC: Huangkui capsules; n = 4, each group, *P < 0.05, **P < 0.01, ***P < 0.001, one-way ANOVA test.

We initially monitored various physiological indices in all studied animals to evaluate the reno-protective efficacy of HKC in DN. The data indicated that the UACR levels in DN group were expectedly higher compared to those in T2D group (Figure 1A). After consecutive 4 weeks of HKC administration, however, UACR and UAER levels in HKC group were significantly reduced (Figure 1A; Supplementary Figure S1A). BG and BW levels among T2D, DN, and HKC groups were analyzed, and no statistically significant change was observed (Figures 1B,C). Furthermore, H&E and PAS staining optical photographs of kidneys demonstrated that the thickened glomerular basement membrane and diffused hyperplasia, glomerular atrophy, and hyaline capillaropathy were presented in DN group while these damages were observed to be decreased in the HKC group (Figure 1D; Supplementary Figure S1B,C).

### 3.2 DNA methylation status in kidneys of DN and its changes after HKC administration

**FIGURE 2:**
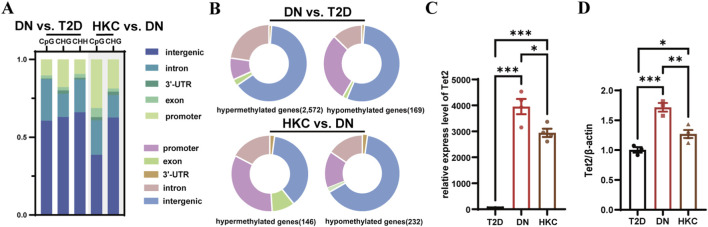
DNA methylation status in kidneys of DN and its changes after the HKC administration. **(A)** Comparison of DNA methylation patterns among groups and differentially methylated regions (DMRs) annotation in genome functional areas (intergenic, intron, 3′-UTR, exon, and promoter). The X-axis represents the 5 mC types annotated to DMRs within each group, while the Y-axis indicates the proportion of 5mCs annotated to various gene elements. **(B)** Distribution of gene element annotations for hypermethylated genes and hypomethylated genes, and the comparative number of hypermethylated and hypomethylated genes in DN vs. T2D and HKC vs. DN. **(C)** Relative expression of DNA methylation-associated gene Tet2 (Ten-Eleven Translocation 2) with normalized counts as a reference. The Y-axis displays the relative express level of Tet2 in RNA-seq. **(D)** Quantitative validation of Tet2 by RT-qPCR, β-actin served as an internal reference. Values are given as mean ± SEM of three or four replicates. *P < 0.05, **P < 0.01, ***P < 0.001, one-way ANOVA test.

We then investigated the DNA methylation status in DN, and its changes after HKC administration by using WGBS. Based on the data of DNA methylation levels (the fraction of methylated cytosine) among T2D, DN, and HKC groups and the principal component and Pearson correlation analyses, the stabilized consistency of DNA methylation levels within each group and the obvious heterogeneity between the groups were found (Supplementary Figure S3A,B). Overall, most of the cytosines in the CpG sites were methylated. Compared to T2D, the DNA methylated sites in DN were annotated in intergenic and intron regions. The comparison of HKC with DN, however, showed that the proportion of DNA methylated sites annotated to exon and promoter regions was increased (Figure 2A), which was consistent with the percentage of methylated cytosines annotated to hypomethylated and hypermethylated genes (Figure 2B). Interestingly, there were multiple distinct methylation patterns for the multi-site in the same gene. Most of the pivotal genes in DN had three distinct types of methylation sites, i.e. CpG, CHG, and CHH while the highly epigenetic susceptibility genes after HKC administration exposure had CpG and CHG methylation patterns but not CHH (Supplementary Figure S1C).

*Tet2* encode the demethylation enzymes, respectively, and play a crucial regulatory role in DNA methylation (Williams et al., 2011). In the current study, the expression of *Tet2* genes was found to be increased in DN. After HKC treatment, the expression was decreased (Figure 2C). Furthermore, the aberrant DNA methylation patterns in DN group were found to be exacerbated with the progression of DN, while HKC may reverse the *Tet2* expression and resulting in affecting the abnormal methylation patterns.

### 3.3 DNA methylation landscapes and gene expression patterns in kidneys after HKC administration

**FIGURE 3:**
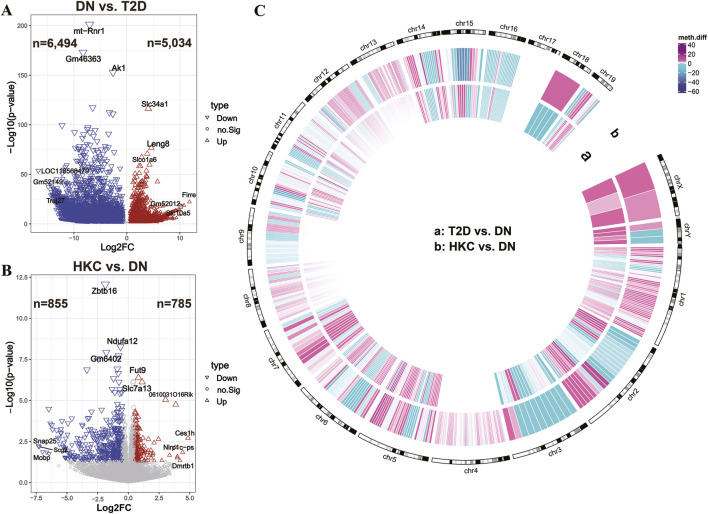
The changes in DNA methylation and gene expression patterns after HKC administration. **(A,B)** Expression pattern volcano plots of differentially expressed genes (DEGs) obtained in each group compared with the DN group (genes with |log2FC| ≥ 0.5 and p-value <0.05 were identified as DEGs), the most significantly altered and most credible genes, along with the number of up genes and down genes, are marked in the figure. The X-axis represents log2 (fold change), while the Y-axis displays the negative logarithm of the gene’s T-test significance *P*-value. **(C)** Distribution of identified DMRs on each chromosome and the color of DMRs represents their methylation levels, the Circos plot with the chromosome numbering and DMR position markers on the outer ring, and the inner ring in the order of CpG in a: T2D vs. DN, b: HKC vs. DN.

We further integrated WGBS and RNA-seq data to analyze gene expression levels in two comparisons: DN vs. T2D and HKC vs. DN. The different gene expression patterns of DN vs. T2D and HKC vs. DN were represented in the utilizing volcano plots (Figures 3A,B). The initiation of DN was accompanied by downregulation of massive genes (n = 6,494). The number of downregulated DEGs (n = 855) was greater than the number of upregulated DEGs (n = 785) after HKC treatment. In Figure 3C, a Circos plot showed that the DMRs in the context of CpG annotated chromosomal distribution were substantially identical, and the changes of methylation regions were evenly distributed across the genome. Overall, the development of DN and HKC administration was accompanied by altered gene methylation patterns as well as fluctuations in gene expression.

### 3.4 Functional characterization of the genes differentially methylated and expressed

**FIGURE 4:**
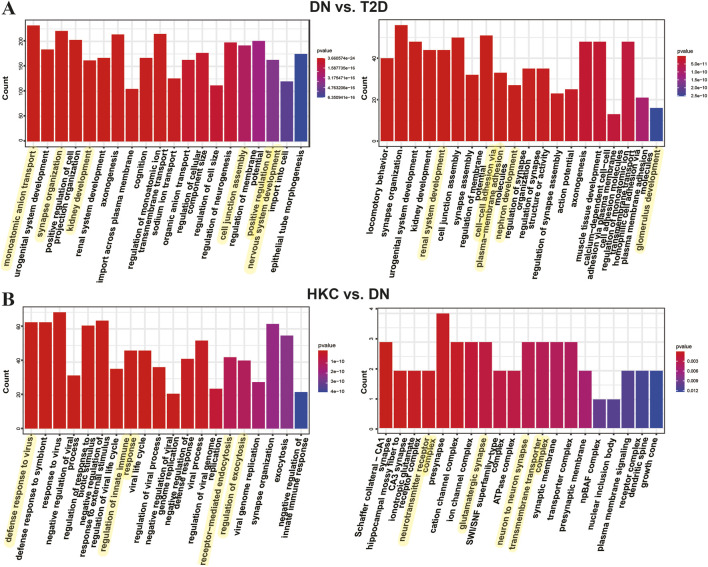
Functional characterization of the genes differentially methylated and expressed. **(A)** Bar graph of GO enrichment analysis of DEGs (left) and differentially methylated genes (DMGs) (right) obtained from DN vs. T2D identification. **(B)** GO enrichment analysis results of DEGs and DMGs obtained from HKC vs. DN identification. The most significantly enriched top 20 pathways of DEGs and DMGs in each group compared with the DN group, and the terms associated with kidney functions are highlighted in yellow.

A total of 3,233 DMGs and 11,528 DEGs were identified in DN vs. T2D, along with 438 DMGs and 1,640 DEGs in HKC vs. DN. The probable biological roles of the differential genes were then elucidated through GO and KEGG pathway enrichment analyses. We found that DEGs in T2D group were mainly enriched in renal and neural development, ion transmembrane transport-relative, PI3K-Akt signaling, focal adhesion, Rap1 signaling, amino acid and fatty acid metabolism pathways (Figure 4A left). Meanwhile, DMGs were mostly involved in cell adhesion and metastasis, renal unit and glomerular formation, PI3K-Akt signaling pathway, MAPK, Ras, and Rap1 signaling pathways (Figure 4A right), in which the cross-talk between PI3K-Akt and MAPK pathway was associated with renal interstitial fibrosis (Zhang et al., 2021). HKC administration-driven DEGs were mainly enriched in biological activities such as immune regulation, viral defense, cytotoxicity, and endocytosis, as well as related to neuroactive receptor-ligand interactions and cytokine interaction pathways, and its DMGs were mainly involved in biological processes such as neural synaptic signaling, ion transmembrane transport, and Notch signaling pathway (Figure 4B). Literature records have demonstrated that Notch signaling regulates nephron number and segmentation (Bonegio and Susztak, 2012), impacting albuminuria, glomerulosclerosis, renal function, and susceptibility to renal disease (Murea et al., 2010). Inhibiting the Notch signaling system is thought to be a novel therapeutic method for DN (Lin et al., 2010).

### 3.5 DNA methylation and gene expression correlation analyses in DN

**FIGURE 5:**
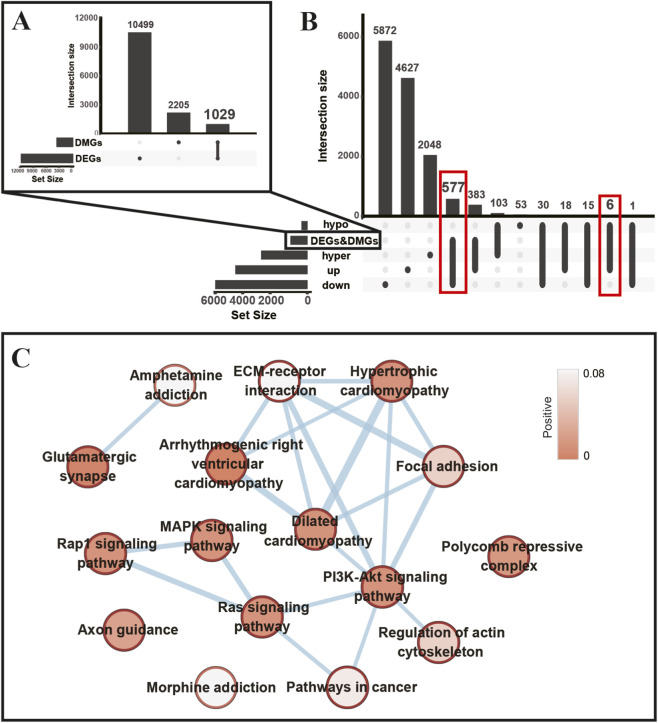
DNA methylation and gene expression correlation analysis for DN causative genes. **(A)** The intersection of DEGs and DMGs from DN vs. T2D identification. **(B)** Comparison of hypermethylated downregulated genes and hypomethylated upregulated genes in DEGs and DMGs from DN vs. T2D and the red box highlights hypermethylated and downregulated genes and hypomethylated and upregulated genes in Upset analysis. hyper: hypermethylated gene, hypo: hypomethylated gene, up: upregulated gene, down: downregulated gene. **(C)** A KEGG pathway enrichment map of potential crucial genes involved in the development of DN.

Finally, we combined RNA-Seq and WGBS data to identify genes with both differential expression and differential methylation, revealing DN-associated methylation candidate genes that may be causal in DN and contribute to the epigenetic mechanisms underlying DN. The results of DN vs. T2D yielded 1,029 genes with one or more methylated sites and significantly altered expression with the development of DN (Figure 5A), and further investigation revealed that 577 genes were downregulated in expression due to hypermethylated sites and 6 hypomethylated upregulated genes (Figure 5B). Supplementary Table S1 shows the expression and methylation sites of 25 crucial genes involved in the methylation status in DN. *Pcdh15, Mdga2, Fmo6*, and *Sp140* demonstrated a strong positive correlation between methylation sites and gene expression (Supplementary Figure S2). We found that hypermethylated downregulated genes were associated with kidney development and ion transport, whereas hypermethylated upregulated genes were mostly connected with biological processes including cell adhesion and synapse formation. We also discovered that numerous intersecting genes were implicated in the neuroactive ligand-receptor interaction, PI3K-Akt signaling network, cAMP signaling pathway, cell adhesion, and Ras signaling pathway. Simultaneously, there is crosstalk among various pathways (Figure 5C), forming a regulatory network strongly linked to the development of DN.

We identified *Ntrk2* as a potential epigenetic susceptibility gene for DN (Supplementary Table S1). A positional candidate genetic study has demonstrated that *Ntrk2* is a glomerular filtration rate variant gene and its biofunction is involved in the MAPK pathway (Thameem et al., 2015). Moreover, *Rbms3* (Klumpers et al., 2022), *Morc1* (Wang et al., 2021), *Cd300lf* (Zhang et al., 2022), and *Arel1* (Rydbirk et al., 2020) are reported to be associated with epigenetic regulation in kidney diseases, while *Aim2* inflammasome has potential pathogenic effects in kidney diseases, including podocyte damage and kidney inflammation (Chung et al., 2021). Podocyte injury may directly contribute to proteinuria, and the mouse podocyte autophagy regulatory protein in which *Gpr137b* may play an important role (Hu et al., 2020).

### 3.6 The target genes of HKC treatment in kidneys

**FIGURE 6:**
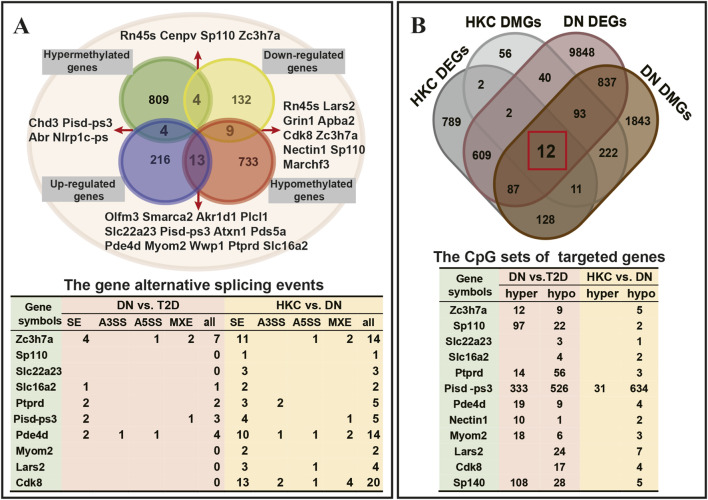
The kidney target genes for Huangkui capsules in the treatment of diabetic nephropathy. **(A)** The genes displayed in the hypermethylated down-related gene set, hypermethylated up-related gene set, hypomethylated up-related gene set, and hypomethylated down-related gene set identified by HKC vs. DN. **(B)** The intersection of DEGs and DMGs obtained from HKC vs. DN identification and DN vs. T2D identification. The tables summarized the changes in alternative splicing events and CpG sites annotations for 12 genes before and after HKC administration.

We further analyzed the genes with changes in methylation and transcript abundance after HKC administration, as shown in Figure 6A and Supplementary Table S2. In total, we identified 28 genes with altered expression due to DNA methylation changes. We comparatively investigated the DNA methylation and transcriptomic expression levels in db/db mice with and without HKC treatment to explore the pharmacological targets of *A. manihot* (L.). As shown in Figure 6B, there are 12 key genes, including *Nectin1, Lars2, Zc3h7a, Cdk8, Slc16a2, Myom2, Slc22a23, Ptprd, Pde4d, Pisd-ps3, Sp140*, and *Sp110* screened by HKC vs. DN. We ultimately identified these 12 genes as epigenetic target genes for the treatment of DN by HKC.

DNA methylation can not only lead to changes in expression, but also cause alternative splicing (AS) during transcription (Jaenisch and Bird, 2003). We identified variable AS of each group as described above using rMATS, combining with the number of CpG methylated sites of genes, we found that HKC administration exposure not only altered the number of methylated sites but also increased the possibility and variability of AS in *Zc3h7a, Pde4d, Cdk8, and Pisd-ps3*, suggesting the high apparent susceptibility of these four genes might be related to the therapeutic mechanism of HKC (Supplementary Figure S4).

## 4 Discussion

In the present study, we aimed to explore the kidney target genes of HKC in the treatment of DN by using WGBS and RNA-seq analyses. Based on the WGBS data, we found that most of the cytosines were methylated in the CpG sites. A total of 3,159 hypermethylated and 225 hypomethylated structural domains were identified in DN vs. T2D. This phenomenon reflected that the changes of DNA methylation patterns in T2D group might exist in the specific methylation-changed genes but not overall. Combined with the specific gene methylation level distribution, the majority of DMRs were found in intergenic regions, while a tiny portion of DMRs was localized in the regions of promoter and exon.

Combined with the data of RNA-seq, we further found that the transcription was activated when hypermethylation occurred in the genes, which could be repressed when hypermethylation occurred in the promoters of genes (Ehrlich and Lacey, 2013). DNMT1/3A are mainly involved in the maintenance of methylation and *ab initio* DNA methylation (Li et al., 2007; Song et al., 2012), while TET2 plays an important role in the elimination of methylation (Williams et al., 2011). In an *in vivo* trial of db/db mice and human mesangial cells (HMCs) experiment, high glucose-induced increased expression of TET2 in the renal cortex and HMCs, and pathological changes in DN were reversed by short hairpin RNA (shRNA) knockdown of *Tet2*, which originated on the demethylation of the CpG island in the *Tgf-β1* regulatory region by *Tet2* (Yang et al., 2018). In further study, Liang et al. found that *Tet2* knockout mice exhibited a significantly reduced risk of kidney disease, with *Tet2* identified as a novel kidney disease risk gene associated with DNA damage repair and nucleotide sensing mechanisms (Liang et al., 2024). Our study found that HKC significantly reduced *Tet2* levels in the kidney, indicating its positive effect on altering the renal methylation status in db/db mice. The pharmacological action of HKC may be related to changes in the expression of renal target genes influenced by *Tet2*’s demethylating activity.

The reno-protective efficacy of HKC is multiple genes targeted (Li et al., 2021). In the present study, we screened 12 key genes based on the integration analysis of DNA methylation and transcriptional profiling. Some of these genes play critical roles in DN and associated with metabolic disorders. For instance, the mitochondrial-specific translation gene *LARS2* has been reported as a novel susceptibility gene for T2D, with its variants associated with the disease risk (‘t Hart et al., 2005). Furthermore, *SLC16A2* participates in multiple metabolic pathways across various tissues, and its epigenetic alterations may impair normal physiological functions. Bansal et al. confirmed that epigenetic changes in this SLC within renal proximal tubule epithelial cells of DN patients correlate with chronic renal insufficiency (Bansal et al., 2020), providing evidence that HKC improves metabolic disorders and renal function through epigenetic pathways (Wang et al., 2025). Furthermore, *Nectin1* may play a role in renal development due to its involvement in renal epithelial cell morphogenesis (Brakeman et al., 2009). *Pde4d* induces renal injury by enhancing hepatorenal interstitial signaling, and plays a role in the hepatorenal axis of DN (Tao et al., 2023).

We then carried out the enrichment analysis. The data revealed that DMGs and DEGs of DN vs. T2D were enriched in nephron and glomerular development, PI3K-Akt signaling pathway, cell adhesion and metastasis, and MAPK signaling pathway. The PI3K-Akt signaling pathway plays a key role in epithelial-mesenchymal transition and podocyte injury in renal tubular cells (Huang et al., 2023). It is unarguably believed that renal sympathetic (efferent) nerves contribute to the regulation of glomerular filtration, sodium reabsorption, and renin release, but far less is known about their contribution to renal disease states previously (Kato and Natarajan, 2014), and renal nerves are hypothesized to potentiate or modulate disease through immune system crosstalk (Osborn et al., 2021) or specific neurotransmitter release (Page and Heuer, 1935).

We have screened several genes in DMGs and DEGs and found that they are related to neuronal development and function and included *Morc1, Grik4, Dlgap2, Npffr1, Npas3, Zfp536, Dlgap1, Mdga2, Galnt13*, and *Ntrk2*. As an epigenetic regulator, *Morc1* is reported to be associated with cancer and neurogenic diseases (Mikami et al., 2013); *Npffr1* is enriched in pro-adrenocorticotropic hormone-releasing hormone neurons and is involved in neuroendocrine (Higo et al., 2021). *Ntrk2* has been identified as a susceptibility gene and is associated with IgA nephropathy (Hahn et al., 2011) and glomerular filtration rate (Thameem et al., 2015). Furthermore, DN is characterized by both endocrine and renal nervous system abnormalities (Barrett et al., 2017). The development of DN is intimately related to neuromodulation and neuro-metabolism. Thereby, the synergistic/regulatory role of the neurological and endocrine systems is indispensable (Irimia Sieira et al., 2019). In the present study, we found several genes such as *Pcdh15*, *Dsc3*, and *Ank2* to be related to cell adhesion, indicating that epigenetic changes alter components in epithelial cells and basement membranes. In addition, *Fmo6* is a gene encoded for basal metabolic functions of kidneys, suggesting that epigenetic modifications may affect renal metabolism.

DNA methylation can influence gene expression and AS without changing the DNA sequence, which results in the alteration of biological processes and subsequently changes in cellular phenotype and function (Jaenisch and Bird, 2003). Specific knockdown of the spliceosome regulator *Srsf7* in the proximal tubule induces a proinflammatory phenotype, and a split phenotype of *VEGFA* plays a role in maintaining the normal function of the glomerular filtration barrier, suggesting that AS is a driver of DN (Stevens and Oltean, 2018; Wu H. et al., 2022). Additional mechanistic insights can be gained from the analysis of data at the exon and isoform level compared to standard gene level analysis: we observed that *Zc3h7a*, *Pde4d, Cdk8,* and *Pisd-ps3* exhibited significant differential splicing in the HKC-administered group compared to the DN group. We speculated that HKC administration altered the overall methylation level of the genome and increased the likelihood of AS of mRNAs. This AS generated the isoform family with different functions, which may be relevant to the reno-protective efficacy of HKC.

In the present study, we have analyzed the reduction of urinary protein levels and renal protection induced by HKC administration from an epigenetic perspective. The data demonstrated that HKC administration induced the changes of DNA methylation and mRNA expression of the kidney target gene of *A. manihot* (L.) in the treatment of DN, while these genes play key roles in PI3k and Notch signal pathways. By using the research approach of the pathway, the previous studies have reported that HKC attenuates the renal tubular epithelial-mesenchymal transition in DN by suppressing NLRP3 inflammasome activation and TLR4/NF-κB signaling and induces mitochondrial autophagy for STING1/PINK1 signaling in renal tubular cells (Han et al., 2019; Zhu et al., 2023).

By using bisulfite sequencing on human whole blood, Li et al. have reported that the overall methylation level of the Foxo1 promoter region decreases progressively with disease progression. This change is closely linked to lipid metabolism in the development of DN (Li et al., 2022). However, this report lacks genome-wide methylation analysis specifically in kidney tissues. Zhao et al. have applied the bulk RNA-seq approach and identified 125 differentially expressed genes associated with DN. These genes are enriched in the pathways related to fatty acid response, macrophage differentiation, and cholesterol metabolism (Zhao et al., 2022a). In the present study, we have performed further analysis of the correlation between DNA methylation and mRNA expression levels based upon the data of methylation genomics and transcriptomics at a whole genome scale. The results from the present study could be better to illustrate the epigenetic role in DN as well as the epi-pharmacological efficacy of HKC in the treatment of DN.

There are a couple of limitations in the present study. First, we have focused on the analysis of DNA methylation (5 mC) but not RNA methylation (m6A) as well, which may cause a limited understanding of the fundamental association between these two nucleic acid modifications. Second, the AS between rodents and humans is susceptible and weakly conserved. Therefore, further investigation of renal target genes of HKC in the treatment of DN by using clinical material in T2D-DN patients has been taken into our consideration.

## 5 Conclusion

This study for the first time explores the epigenetic pharmaceutical effects of HKC in the treatment of DN. The data also provide a comprehensive DNA methylation profile of kidneys in DN and reveal the epigenetic pharmaceutical effects of HKC on the target genes in kidneys. Further investigation with biological experiments to validate these findings at protein levels has been taken into our consideration.

## Data Availability

The original contributions presented in the study are publicly available. This data can be found here: NCBI repository, accession number PRJNA945213.

## Glossary

A3SS: alternative 3′splice sites
A5SS: alternative 5′-splice sites
AS: alternative splicing
BG: blood glucose
BW: body weight
Cr: creatinine
DEG: differentially expressed genes
DMG: differentially methylated gene
DMR: differentially methylated region
DMS: differentially methylated site
DN: diabetic nephropathy
Down genes: downregulated genes
ESRD: end-stage renal disease
GO: Gene ontology
H&E staining: Hematoxylin and Eosin staining
HKC: Huangkui capsule
HMCs: human mesangial cells
Hyper genes: hypermethylated genes
Hypo genes: hypomethylated genes
KEGG: Tokyo Encyclopedia of Genes and Genomes
MAU: microscale albuminuria
MXE: mutually exclusive exons
RI: retained intron
RNA-Seq: RNA transcriptome sequencing analysis
scWGBS: single-cell whole genome bisulfite sequencing
SE: exon skipping
shRNA: short hairpin RNA
T2D: type 2 diabetes
TCPM: traditional Chinese patent medicine
PAS staining: Periodic Acid-Schiff staining
UACR: urinary albumin/creatinine ratio
UAER: urine albumin excretion rate
Up genes: upregulated genes
WGBS: whole genome bisulfite sequencing
