# Supplementary material for: Huangkui capsule mitigates diabetic nephropathy via epigenetic therapy effects
Source: Front Pharmacol. 2026 Feb 24;17:1775173. doi: 10.3389/fphar.2026.1775173 (PMC12972618; doi:10.3389/fphar.2026.1775173)
Supplement: Supplementary file 1 [file DataSheet1.pdf]

Supplemental information

Yu et al. Huangkui capsules ameliorate diabetic nephropathy by affecting epigenetic effects

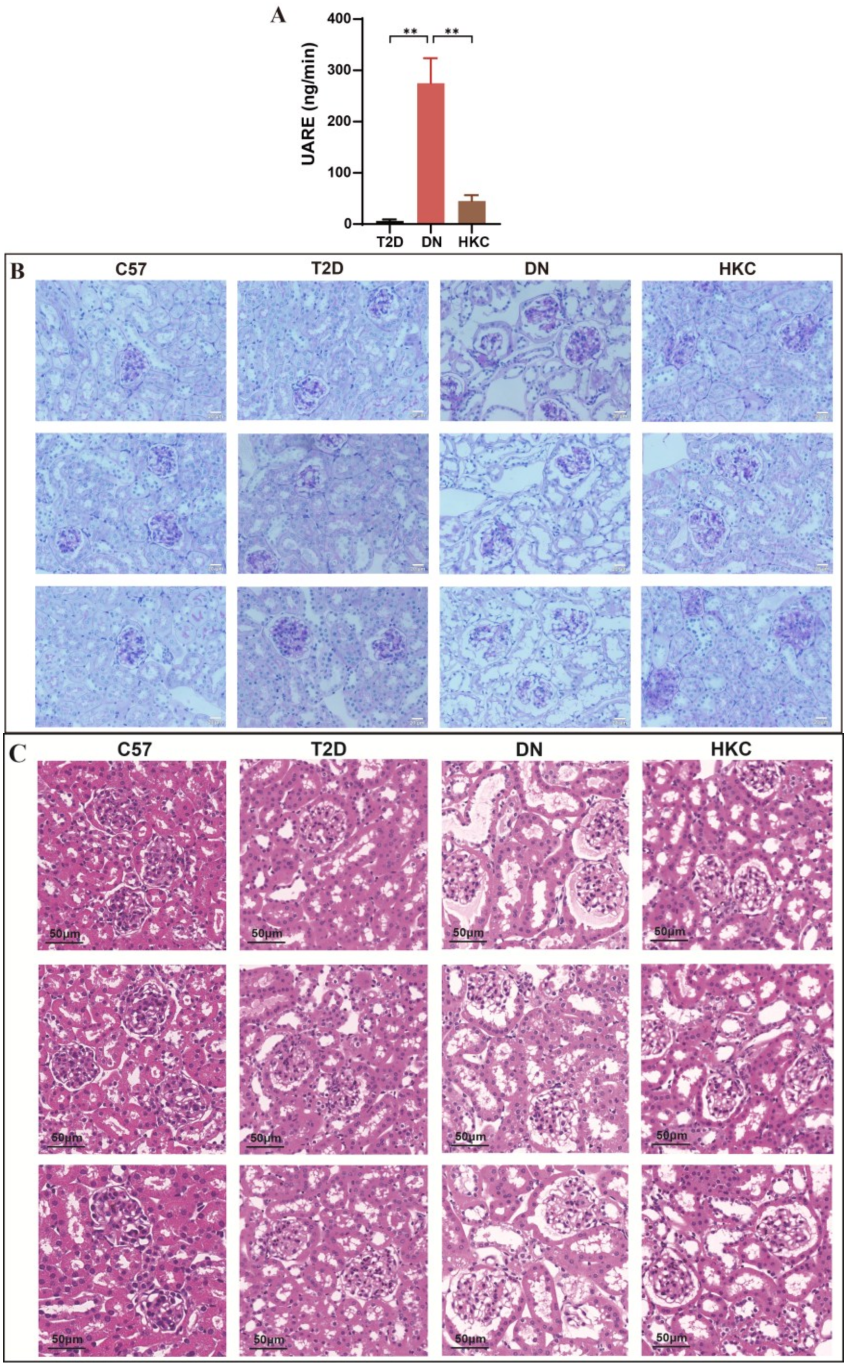

Figure S1 (A) UAER levels in the groups of T2D, DN and HKC. UAER: Urine Albumin Excretion Rate; T2D: type 2 diabetes; DN: diabetic nephropathy; HKC: Huangkui capsule treatment. * $P < 0.05$, ** $P < 0.01$, *** $P < 0.001$, one-way ANOVA test. (B) The images of PAS-stained kidney tissue sections in C57, T2D, DN and HKC groups show glomerular injury and hyaline capillaropathy in the DN group, while these damages were observed to be decreased in HKC group. Scale bars = 20 μm. (C) H&E-stained kidney tissues images from each group of mice. Scale bars = 50 μm.

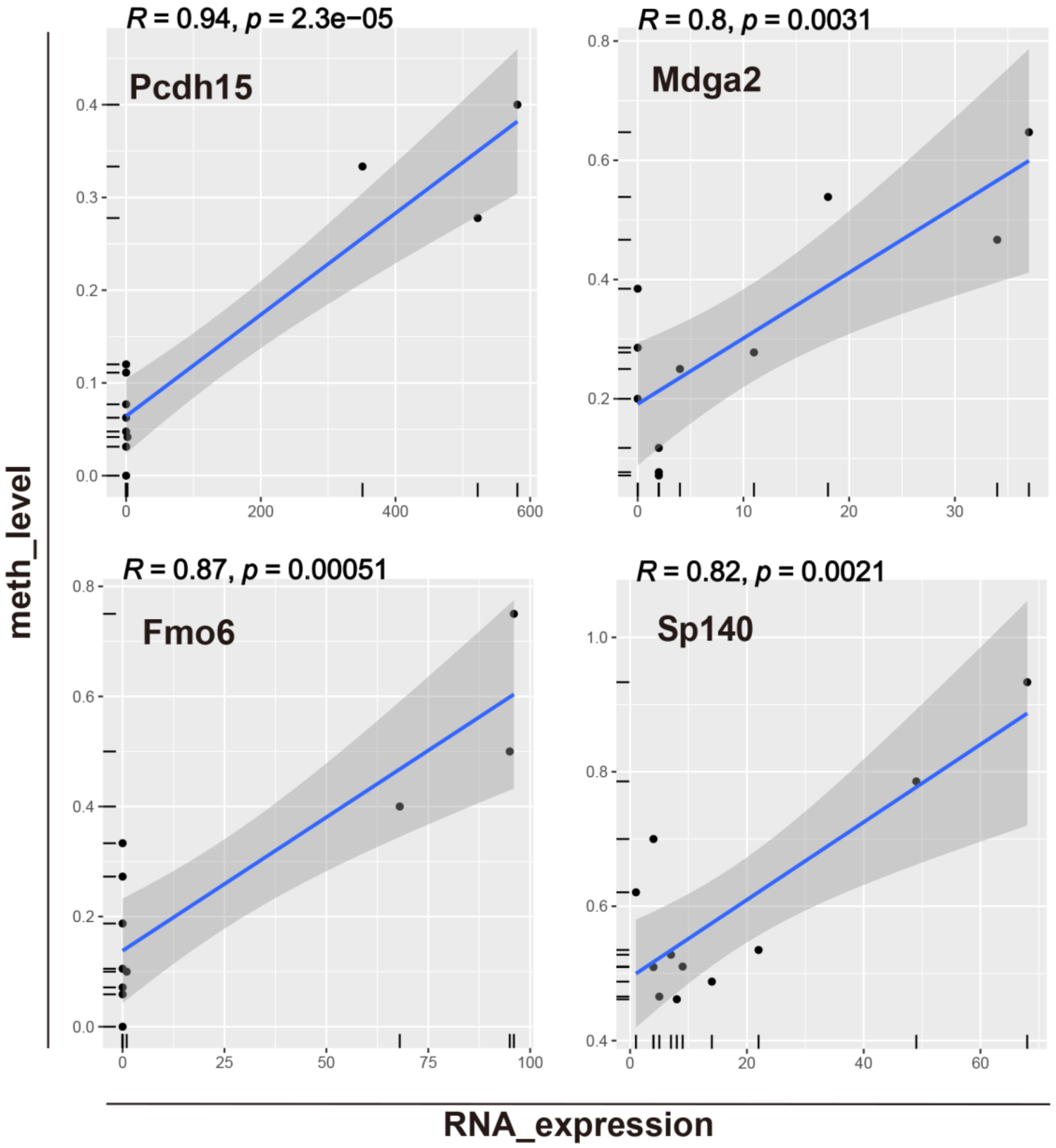

Figure S2 The correlation analysis between DNA methylation levels and RNA expressions of the DN target genes. DMSs of the *Pcdh15* (chr1 73772202-73772202), *Mdga2* (chr12 67177042-67177042), *Fmo6* (chr1 162928211-162928211), and *Sp140* (chr1 85104683-85104683) genes showed a strong positive correlation with gene expression, parentheses describe the chromosomes, start and end loci of the DMS, meth_level: methylation levels of DMSs, RNA_exp: RNA expression of DEGs by normalized counts.

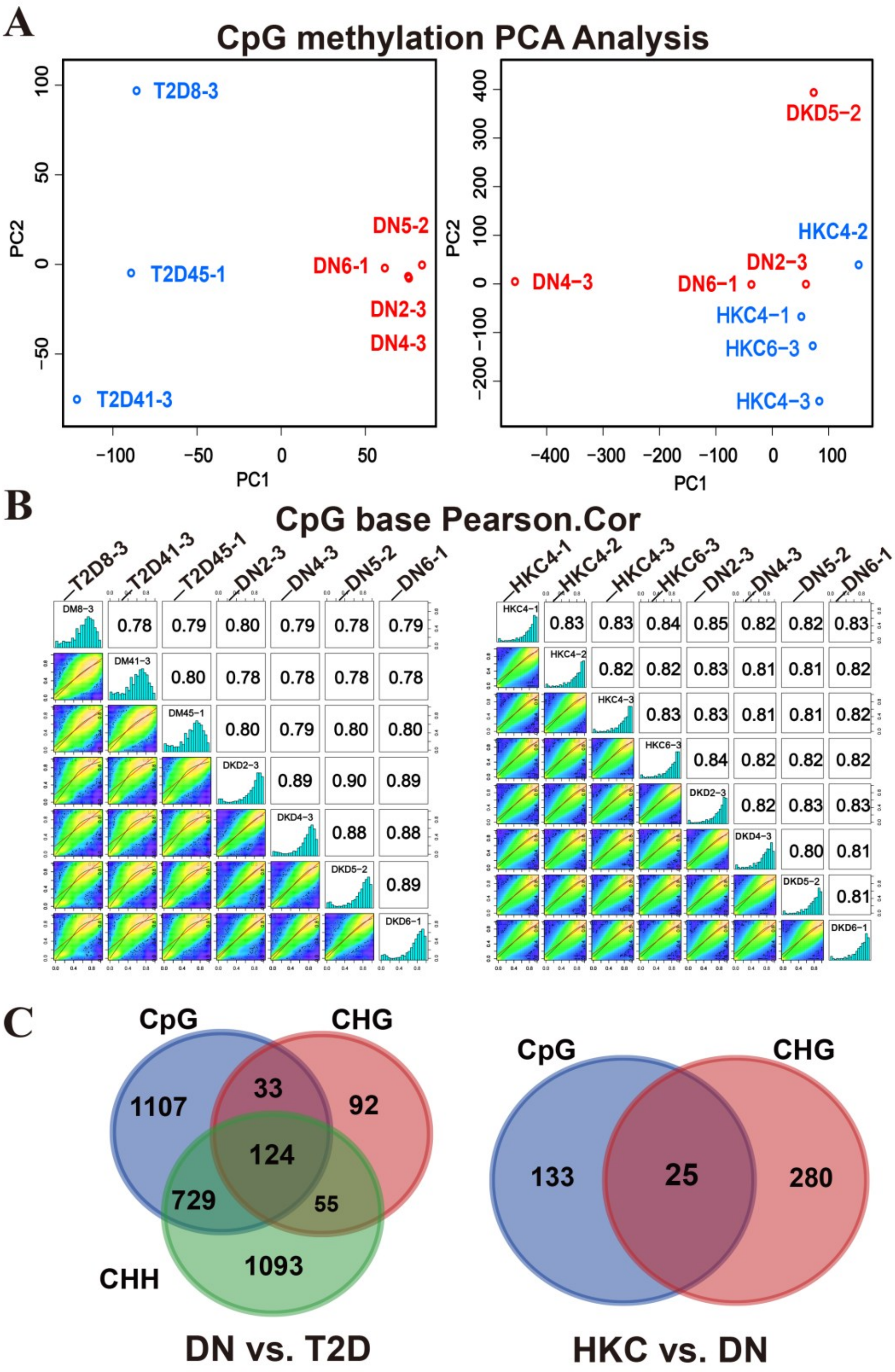

Figure S3 (A) PCA analysis based on CpG methylation between groups. Different groups were labeled with different colors; (B) Methylation correlation analysis among groups; The heat map displays the distribution of methylated CpG sites, while the bar graph displays their frequency and (C) Venn plot of DMGs identified in different contexts.

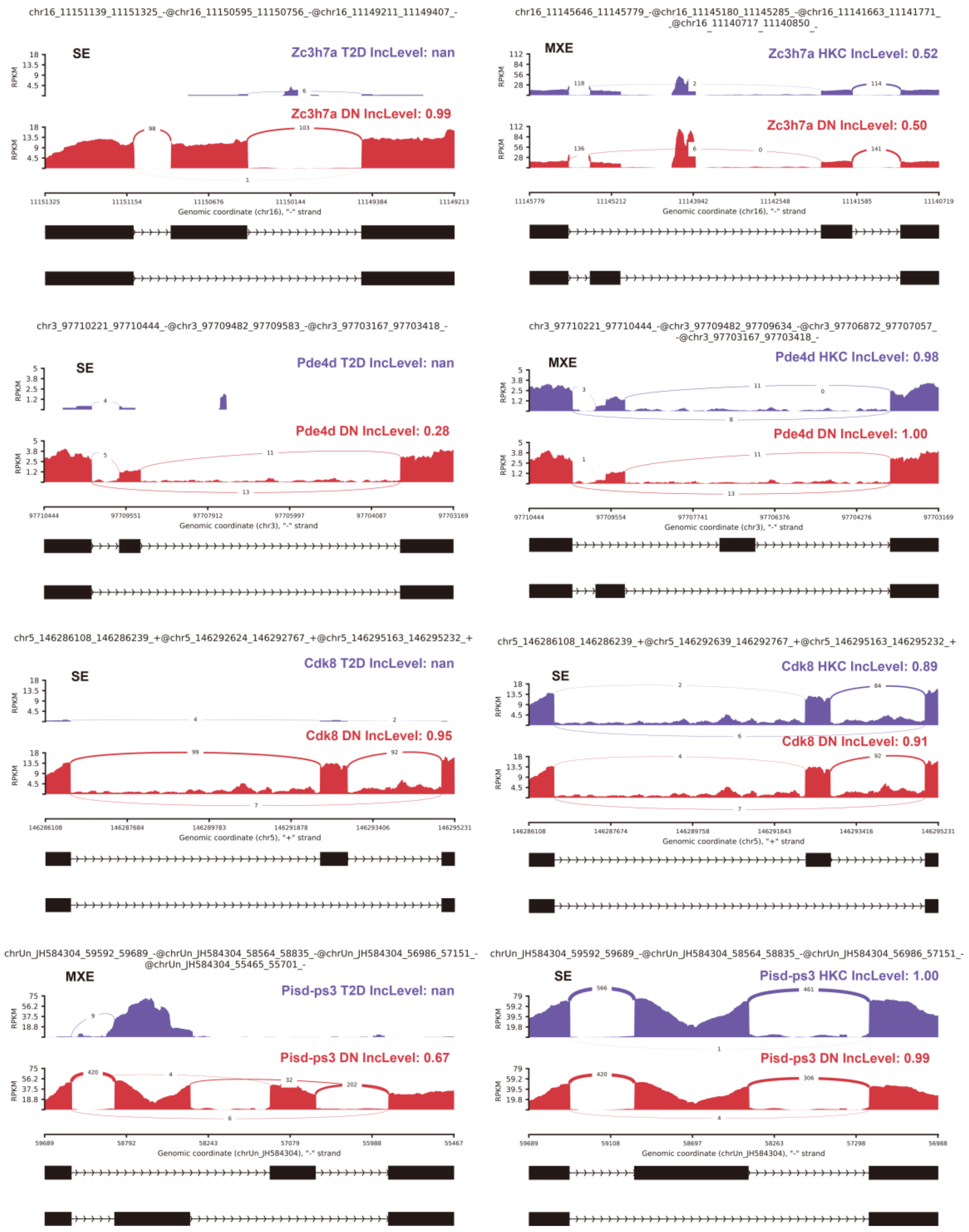

Figure S4 The alternative splicing of the *Zc3h7a, Pde4d*, *Cdk8*, and *Pisd-ps3* genes. The AS type of the genes is seen in the upper left corner of the images.

Table S1 The genes and their DNA methylation and mRNA expression changes in the kidneys of db/db mice with diabetic nephropathy

Gene symbol	Gene name	DNA methylation difference (DN vs T2D)		RNA Expression (DN vs T2D)	Biofunction	Related to kidney disease/UACR
		Methylation difference	Type	log2FC		
Dlgap1	DLG-associated protein 1	0.389/0.631/0.631/70.238/34.337/27.063/	intron	-6.275	A molecular adaptor, a structural constituent of postsynaptic density.	NA
Mdga2	MAM domain containing glycosylphosphatidylinositol anchor 2	31.165/69.156/	intron	-6.269	Involved in the regulation of presynaptic assembly, synaptic membrane adhesion, neuronal migration, and synaptic function.	NA
Kcnh8	potassium voltage-gated channel subfamily H member 8	1.009/34.172/	intron	-6.943	A member of the subfamily of potassium voltage-gated channels.	NA
Pisd-ps3	phosphatidylserine decarboxylase, pseudogene 3	-6.888/30.384 #	promoter/exon/intron/3'-UTR	-1.306	A pseudogene, expressed in the nervous system and olfactory epithelium, is a new candidate gene for obesity association (Mikec et al., 2023).	NA
Ank2	ankyrin 2	1.275/0.662/0.549/1.336/1.336/34.895/55.000/	intron	-1.012	An anchor protein, related to cell activities.	NA
Galnt13	polypeptide N-	0.945/0.704/0.734/0.811/0.945/0.935/0.889/0.913/34.	intron	-5.328	A UDP-N-acetyl-alpha-D-galactosamine may promote neurogenesis (Xu et al.,	NA

	acetylgalactosaminyltransferase 13	615/40.082/54.765/			2016).	
Gng10	G protein subunit gamma 10	51.208	intron	-1.443	It is involved in the G protein-coupled receptor signaling pathway.	NA
Ntrk2	neurotrophic receptor tyrosine kinase 2	0.356<br>50.893	intron<br>3'-UTR	-4.042	A neurotrophic tyrosine receptor kinase, is involved in the MAPK pathway.	*Ntrk2* is a susceptibility gene for childhood IgA nephropathy (Hahn et al., 2011). Genetic variation in glomerular filtration rate susceptibility genes (Thameem et al., 2015).
Rbms3	RNA binding motif single stranded interacting protein 3	0.620/25.238/48.998/33.644/33.396/	intron	-2.797	An RNA-binding protein that belongs to the c-myc gene single-strand binding protein family.	There are associations between genetic variants in *RBMS3* and acute kidney injury phenotypes (Klumpers et al., 2022).
Aim2	absent in melanoma 2	33.714/48.504/	intron	-3.653	A member of the IFI20X /IFI16 family that plays a putative role in tumorigenic reversion and may control cell proliferation.	AIM2 inflammasome has potential pathogenic effects in kidney diseases (Komada et al., 2018). Aim2(-/-) (B6) mice exhibited podocyte loss and proteinuria (Chung et al., 2021).
Pcdh15	protocadherin related 15	0.426/0.470/0.492/0.493/28.680/26.111/33.944/48.707/30.099/<br>0.719	intron<br>3'-UTR	-13.168	A calcium-dependent integral membrane protein.	NA
Morc1	MORC family CW-	0.455/27.497/	intron	-12.379	An epigenetic regulator and chromatin remodeling factor in germ cell	NA

	type zinc finger				development.	
Fmo6	flavin containing monooxygenase	47.262	intron	-12.139	A transmembrane protein of the endoplasmic reticulum of renal cells with monooxygenase activity.	NA
Npffr1	neuropeptide FF receptor 1	0.703/0.758/0.709/	intron	-12.010	A G protein-coupled receptor.	NA
Grik4	glutamate ionotropic receptor kainate type subunit 4	0.639/30.556	intron	-11.780	An excitatory neurotransmitter.	NA
Dlgap2	DLG associated protein 2	0.765027322<br>27.13032581	promoter<br>intron	-6.275	A membrane-associated protein that may play a role in synapse organization and signaling in neuronal cells.	NA
Otud7a	OTU deubiquitinase 7A	0.105/0.154/0.490/0.564/	intron	-10.963	A deubiquitinizing enzyme and possible tumor suppressor that acts on TNF receptor-associated factor 6 (TRAF6) to control nuclear factor kappa B expression.	NA
Npas3	neuronal PAS domain protein 3	0.149/0.406/0.465/	intron	-10.900	A member of the basic helix-loop-helix and PAS domain-containing family of transcription factors.	NA
Zfp536	zinc finger protein 536	27.818/30.058/38.497/42.152/	intron	-10.619	Enables retinoic acid-responsive element binding activity. Acts upstream of or within negative regulation of neuron differentiation and negative regulation of retinoic acid receptor signaling pathway.	NA
Loxhd1	lipoxygenase	0.361/0.362/0.346/	intron	-10.469	A highly conserved protein consisting	NA

	homology PLAT domains 1				entirely of PLAT (polycystin/lipoxygenase/alpha-toxin) domains, thought to be involved in targeting proteins to the plasma membrane.	
Cd300lf	CD300 molecule like family member f	-28.479	intron	4.293	A CD300 protein.	A high-fat, high-sugar, high-salt diet may lead to methylation of the CpG island in the promoter of the Cd300lf gene, and its F2 female offspring have reduced Cd300lf mRNA expression, increased urinary albumin-creatinine ratios, and an increased likelihood of developing CKD (Zhang et al., 2022).
Rad23b	RAD23 homolog B	-1.712/-1.586/	intron	0.738	A nucleotide excision repair protein.	NA
Arel1	apoptosis resistant E3 ubiquitin protein ligase 1	-0.717/-0.673/-0.558/	promoter	3.304	It enables ubiquitin-protein transferase activity.	NA
Gpr137b	G protein-coupled receptor 137B	-0.426	intron	2.332	It is involved in TORC1 signaling.	*Gpr137b* encodes mice podocyte autophagy regulatory protein upregulated during kidney development (Hu et al., 2020).
Rab37	Rab37 member RAS oncogene	-0.268/-0.283/	distal intergenic	4.158	A RAS oncogene, a key regulator of vesicle trafficking.	Specific knockdown of the *Rab37* gene resulted in a significant reduction in renal

family	cancer cell growth, and the TMEM22/RAB37 complex may play a key role in the growth of renal cancer cells (Dobashi et al., 2009).

Table S2 The genes and their DNA methylation and mRNA expression changes in the kidneys after Huangkui capsule administration

Gene symbol	Gene name	db/db mice with DN [a]			After HKC treatment [b]			Biological function	Related with proteinuria
		DNA methylation	region	mRNA expression	DNA methylation	region	mRNA expression		
Cenpv	Centromere protein V	NA	NA	0.641	25.862	intron	-0.439	Involved in pericentric heterochromatin assembly, positive regulation of cytokinesis; and regulation of chromosome organization.	NA
Rn45s	45S pre-ribosomal RNA	-28.511/3.604/#	promoter	NA	-3.604//22.136/	promoter	0.186	A rRNA serves as the precursor for the 18S, 5.8S, and 28S rRNA.	*Rn45s* expression was significantly reduced in pancreatic islets, which may be related to the pre-diabetic state of T2D (Neelankal John et al., 2018).
Sp110	SP110 nuclear body protein	0.527/25.377/25.460/34.593/<br>0.461/0.596/<br>0.471/0.608/	promoter<br>exon<br>intron	5.566	4.704/-0.494/	intron	-0.641	The protein can function as an activator of gene transcription and may serve as a nuclear hormone receptor coactivator.	NA
Zc3h7a	Zinc finger CCCH-type containing 7A	-3.675/-2.230/-1.914/-1.837/-1.180/-0.852/0.928/1.015/1.167/1.284/1.388/1.937/2.242/2.250/2.262/2.460/2.478/	promoter	1.974	-2.478/-2.460/-2.242/-1.388/-0.928/2.141/7.723/9.328/9.500/12.485/13.639/14.229/14.988/25.445/25.767/25.901/26.626/26.726/26.843/27.234/27.315/27.328/27.383/27.526/27.633/27.797/27	promoter	-0.253	Enables miRNA binding activity. production of miRNAs involved in gene silencing is associated with the regulation of macrophage activation。	NA

					.869/28.456/28.738/28.814/28.880/29.058/29.259/29.274/29.659/29.716/29.769/				
Pisd-ps3	Phosphatidylserine decarboxylase, pseudogene 3	-6.888/-6.017/-5.857/-5.185/-5.147/-5.108/-4.805/-3.942/-3.759/-3.406/-3.262/-2.720/-1.557/-1.519/-0.394/0.138/0.143/0.145/0.155/0.161/0.163/0.169/0.170/0.174/0.177/0.184/0.186/0.188/0.193/0.196/0.200/0.204/0.205/0.206/0.214/0.215/0.216/0.216/0.217/0.217/0.223/0.225/0.226/0.226/0.230/0.231/0.233/0.233/0.234/0.234/0.234/0.236/0.241/0.245/0.247/0.248/0.248/0.249/0.253/0.254/0.254/0.254/0.260/0.262/0.264/0.267/0.269/0.269/0.273/0.274/0.274/0.275/0.276/0.277/0.278/0.279/0.285/0.286/0.287/0.293/0.293/0.299/0.307/0.314/0.319/0.319/0.319/0.320/0.320/0.324/0.324/0.327/0.327/0.334/0.335/0.337/0.346/0.349/0.354/0.359/0.362/0.364/0.373/0.389/0.396/0.433/0.460/0.557/0.563/0.574/0.586/0.606/0.609/0.653/0.821/0.910/7.750/9.420/10.054/10.822/11.105/29.829/30.384/	promoter	-1.306	-0.910/-0.821/-0.653/-0.609/-0.563/-0.396/-0.389/-0.373/-0.359/-0.335/-0.327/-0.327/-0.320/-0.319/-0.319/-0.307/-0.299/-0.293/-0.276/-0.269/-0.267/-0.264/-0.262/-0.249/-0.248/-0.248/-0.193/-0.170/0.394/	promoter	0.399	A pseudogene, expressed in the nervous system and olfactory epithelium, is a new candidate gene for obesity association.	NA
		0.163/0.168/0.204/0.246/0.270/0.329/	exon		-0.329/-0.270/-0.246/	exon			
		-0.600/-0.465/-0.405/-0.248/-0.244/-0.233/-0.194/-	3'-UTR		-0.323/-0.282/-0.267/-0.202/	intron			

		0.143/0.124/0.139/0.141/0.143/ 0.144/0.150/0.151/0.152/0.152/ 0.155/0.155/0.158/0.167/0.167/ 0.170/0.174/0.180/0.181/0.187/ 0.188/0.195/0.197/0.202/0.206/ 0.207/0.209/0.214/0.217/0.220/ 0.227/0.229/0.233/0.239/0.241/ 0.242/0.244/0.253/0.261/0.269/ 0.269/0.272/0.281/0.291/0.297/ 0.299/0.306/0.307/0.317/0.323/ 0.331/0.332/0.333/0.359/0.363/ 0.373/0.381/0.402/0.402/0.437/ 0.439/			-0.439/-0.437/-0.402/- 0.402/-0.381/-0.373/- 0.363/-0.359/-0.333/- 0.323/-0.307/-0.306/- 0.299/-0.291/-0.253/- 0.244/-0.239/-0.233/- 0.227/- 0.217/0.405/0.465/0.600/	3'- UTR			
Cdk8	Cyclin dependent kinase 8	2.316/4.204/ 5.122 / 5.893 / 9.120 / 9.813 / 10.935 / 12.729 / 15.725 / 16.749 / 16.751 / 18.758 / 24.204 / 25.202 / 25.331 / 25.578/	intro n	1.636	-18.758/-12.729/-9.813/- 5.893/	intro n	-0.200	A cell cycle protein-dependent protein kinase is an important regulator of cell cycle progression.	Participation in TGF-β1-induced upregulation of α-SMA, type I collagen, and fibronectin in primary renal fibroblasts as a mechanism of experimental renal fibrosis (Qu et al., 2014).
Grin1	Glutam ate ionotro pic recepto r NMDA type subunit	-0.555/-0.435/0.418/0.674/	pro mote r	2.408	-0.674/-0.418/	pro mote r	-3.195	A member of the glutamate receptor channel superfamily plays a key role in the plasticity of synapses.	Liu, Y.et, al. pointed out that GRIN1 is a novel potential protein target of dapagliflozin against renal interstitial fibrosis (Liu et al., 2023) and GRIN1 is involved in mice AKI

	1								through uncoupling of NMDA receptors leading to apoptosis and necrosis (Husi et al., 2013).
Lars2	Leucyl-tRNA synthetase 2, mitochondrial	3.451/5.491/5.574/5.920/5.924/6.623/6.816/6.996/8.063/8.246/8.257/9.492/10.024/10.511/10.840/11.171/11.625/11.694/12.767/13.765/	3'-UTR	-8.043	-12.767/-11.171/-10.840/-9.492/-8.257/-5.491/-3.451/	3'-UTR	-0.386	A class 1 aminoacyl-tRNA synthetase, mitochondrial leucyl-tRNA synthetase.	Sequencing of 25 subjects with T2D demonstrates that the *LARS2* gene may be a T2D susceptibility gene ('t Hart et al., 2005), and no kidney disease-related studies reported.
Nectin1	Nectin cell adhesion molecule 1	0.775	exon	2.623	-0.689/-0.529/	distal intergenic	-0.498	An adhesion protein, related to adherens junctions and tight junctions in epithelial and endothelial cells.	Expressed at early stages of nephrogenesis and plays a role in renal epithelial cell morphogenesis, affecting mesenchymal-epithelial transformation (Brakeman et al., 2009).
Atxn1	Ataxin 1	1.023	intron	0.581	-1.023	intron	0.567	Related to autosomal dominant cerebellar ataxias.	NA
Myom2	Myomesin 2	0.32	intron	2.853	-1.835/-1.753/-1.708/	distal intergenic	0.286	Enables actin filament binding activity and kinase binding activity, which acts upstream of or within muscle contraction.	NA

Pde4d	Phosphodiesterase 4D	28.848<br>0.479/0.515/0.638/0.857/	3'-UTR<br>intron	-1.136	-0.857/-0.638/-0.515/-0.479/	intron	0.387	A protein that has 3',5'-cyclo-AMP phosphodiesterase activity and degrades cAMP.	*Pde4d* knockout ameliorates kidney injury in high-fat-fed mice (Lu et al., 2021).
Ptprd	Protein tyrosine phosphatase receptor type D	0.411/0.411/0.583/0.583/0.583/0.697/0.697/0.821/29.685/43.985/	intron	1.371	-0.821/-0.697/-0.697/	intron	0.353	A D-type protein tyrosine phosphatase receptor is involved in intercellular adhesion, nervous system development, and regulation of synapse assembly.	DY et al. pointed out that the polymorphism of *PTPRD* is associated with the risk of clear cell renal cell carcinoma (Du et al., 2013).
Slc16a2	Solute carrier family 16 member 2	0.482/0.594/	intron	3.066	-0.594/-0.482/	intron	0.276	Served as a thyroid hormone transporter protein and is related to the central nervous system.	Epigenetic changes in *SLC16A2* in renal proximal tubular epithelial cells in patients with T2D are related to CTCF, which may contribute to persistent renal insufficiency in DN (Bansal et al., 2020).
Wwp1	WW domain containing E3 ubiquitin protein ligase 1	0.494	intron	0.373	-0.494	intron	0.439	A family of NEDD4-like proteins, which are E3 ubiquitin-ligase molecules and regulate key trafficking decisions, including the targeting of proteins to proteosomes or lysosomes.	NA

The related references

'Hart, L.M., Hansen, T., Rietveld, I., Dekker, J.M., Nijpels, G., Janssen, G.M., et al. (2005). Evidence that the mitochondrial leucyl tRNA synthetase (LARS2) gene represents a novel type 2 diabetes susceptibility gene. *Diabetes* 54(6)**,** 1892-1895. doi: 10.2337/diabetes.54.6.1892.

Bansal, A., Balasubramanian, S., Dhawan, S., Leung, A., Chen, Z., and Natarajan, R. (2020). Integrative Omics Analyses Reveal Epigenetic Memory in Diabetic Renal Cells Regulating Genes Associated With Kidney Dysfunction. *Diabetes* 69(11)**,** 2490-2502. doi: 10.2337/db20-0382.

Brakeman, P.R., Liu, K.D., Shimizu, K., Takai, Y., and Mostov, K.E. (2009). Nectin proteins are expressed at early stages of nephrogenesis and play a role in renal epithelial cell morphogenesis. *Am J Physiol Renal Physiol* 296(3)**,** F564-574. doi: 10.1152/ajprenal.90328.2008.

Chung, H., Komada, T., Lau, A., Chappellaz, M., Platnich, J.M., de Koning, H.D., et al. (2021). AIM2 Suppresses Inflammation and Epithelial Cell Proliferation during Glomerulonephritis. *J Immunol* 207(11)**,** 2799-2812. doi: 10.4049/jimmunol.2100483.

Dobashi, S., Katagiri, T., Hirota, E., Ashida, S., Daigo, Y., Shuin, T., et al. (2009). Involvement of TMEM22 overexpression in the growth of renal cell carcinoma cells. *Oncol Rep* 21(2)**,** 305-312.

Du, Y., Su, T., Tan, X., Li, X., Xie, J., Wang, G., et al. (2013). Polymorphism in protein tyrosine phosphatase receptor delta is associated with the risk of clear cell renal cell carcinoma. *Gene* 512(1)**,** 64-69. doi: 10.1016/j.gene.2012.09.094.

Hahn, W.H., Suh, J.S., and Cho, B.S. (2011). Linkage and association study of neurotrophins and their receptors as novel susceptibility genes for childhood IgA nephropathy. *Pediatr Res* 69(4)**,** 299-305. doi: 10.1203/PDR.0b013e31820b9365.

Hu, Y., Wang, Y., Zhang, X., Jin, X., Pei, W., Wang, L., et al. (2020). TM7SF1, an important autophagy regulatory protein in mouse podocytes. *Biochem Biophys Res Commun* 528(1)**,** 213-219. doi: 10.1016/j.bbrc.2020.05.004.

Husi, H., Sanchez-Niño, M.D., Delles, C., Mullen, W., Vlahou, A., Ortiz, A., et al. (2013). A combinatorial approach of Proteomics and Systems Biology in unravelling the mechanisms of acute kidney injury (AKI): involvement of NMDA receptor GRIN1 in murine AKI. *BMC Syst Biol* 7**,** 110. doi: 10.1186/1752-0509-7-110.

Klumpers, M.J., Witte, W., Gattuso, G., Schiavello, E., Terenziani, M., Massimino, M., et al. (2022). Genome-Wide Analyses of Nephrotoxicity in Platinum-Treated Cancer Patients Identify Association with Genetic Variant in RBMS3 and Acute Kidney Injury. *J Pers Med* 12(6). doi: 10.3390/jpm12060892.

Komada, T., Chung, H., Lau, A., Platnich, J.M., Beck, P.L., Benediktsson, H., et al. (2018). Macrophage Uptake of Necrotic Cell DNA Activates the AIM2 Inflammasome to Regulate a Proinflammatory Phenotype in CKD. *J Am Soc Nephrol* 29(4)**,** 1165-1181. doi: 10.1681/asn.2017080863.

Liu, Y., Wang, Y., Chen, S., Bai, L., Li, F., Wu, Y., et al. (2023). Glutamate ionotropic receptor NMDA type subunit 1: A novel potential protein target of dapagliflozin against renal interstitial fibrosis. *Eur J Pharmacol* 943**,** 175556. doi: 10.1016/j.ejphar.2023.175556.

Lu, J., Qian, C., Ji, Y., Bao, Q., and Lu, B. (2021). Gene Signature Associated With Bromodomain Genes Predicts the Prognosis of Kidney Renal Clear Cell Carcinoma. *Front Genet* 12**,** 643935. doi: 10.3389/fgene.2021.643935.

Mikec, Š., Horvat, S., Wang, H., Michal, J., Kunej, T., and Jiang, Z. (2023). Differential alternative polyadenylation response to high-fat diet between polygenic obese and healthy lean mice. *Biochem Biophys Res Commun* 666**,** 83-91. doi: 10.1016/j.bbrc.2023.05.005.

Neelankal John, A., Ram, R., and Jiang, F.X. (2018). RNA-Seq Analysis of Islets to Characterise the Dedifferentiation in Type 2 Diabetes Model Mice db/db. *Endocr Pathol* 29(3)**,** 207-221. doi: 10.1007/s12022-018-9523-x.

Qu, X., Li, X., Zheng, Y., Ren, Y., Puelles, V.G., Caruana, G., et al. (2014). Regulation of renal fibrosis by Smad3 Thr388 phosphorylation. *Am J Pathol* 184(4)**,** 944-952. doi: 10.1016/j.ajpath.2013.12.003.

Thameem, F., Voruganti, V.S., Blangero, J., Comuzzie, A.G., and Abboud, H.E. (2015). Evaluation of neurotrophic tyrosine receptor kinase 2 (NTRK2) as a positional candidate gene for variation in estimated glomerular filtration rate (eGFR) in Mexican American participants of San Antonio Family Heart study. *J Biomed Sci* 22(1)**,** 23. doi: 10.1186/s12929-015-0123-5.

Xu, Y., Pang, W., Lu, J., Shan, A., and Zhang, Y. (2016). Polypeptide N-Acetylgalactosaminyltransferase 13 Contributes to Neurogenesis via Stabilizing the Mucin-type O-Glycoprotein Podoplanin. *J Biol Chem* 291(45)**,** 23477-23488. doi: 10.1074/jbc.M116.743955.

Zhang, X., Hasan, A.A., Wu, H., Gaballa, M.M.S., Zeng, S., Liu, L., et al. (2022). High-fat, sucrose and salt-rich diet during rat spermatogenesis lead to the development of chronic kidney disease in the female offspring of the F2 generation. *Faseb j* 36(4)**,** e22259. doi: 10.1096/fj.202101789RR.
